# Effect of Keeping Caspar Retractor During Prestige LP Cervical Disc Implantation on Intra‐ and Postoperative Outcomes

**DOI:** 10.1111/os.70438

**Published:** 2026-09-24

**Authors:** Xiaoqiang Zhao, Xiao Yang, Minghe Yao, Kangkang Huang, Tingkui Wu, Shihao Chen, Yi Deng, Hao Liu, Beiyu Wang

**Affiliations:** ^1^ Department of Orthopedic Surgery, West China Hospital Sichuan University Chengdu Sichuan China

**Keywords:** Caspar retractor, cervical disc arthroplasty, cervical spine, implant height, Prestige LP

## Abstract

**Objectives:**

Implantation of the Prestige LP prosthesis requires substantial endplate modification and insertion forces, yet the manufacturer's guide recommends relaxing or completely removing the Caspar retractor before implantation, which we observed to cause vertebral instability during endplate preparation and prosthesis insertion. This study aimed to evaluate the effect of retaining the Caspar retractor during Prestige LP cervical disc implantation.

**Methods:**

A total of 55 patients who underwent cervical disc arthroplasty (CDA) between January and December 2025 were retrospectively reviewed. The retractor group maintained the retractor during endplate preparation and prosthesis implantation, while the no‐retractor group removed it after discectomy. Clinical outcomes and radiographic parameters were assessed preoperatively, intraoperatively, and at 1 week, 3 months, and 6 months postoperatively. Statistical analyses were performed using two‐way repeated‐measures ANOVA, paired and independent *t*‐tests, Mann–Whitney *U* test, and Chi‐square test.

**Results:**

Clinical outcomes were similar in both groups. The retractor group demonstrated superior intraoperative stability with reduced functional spinal unit (FSU) Cobb angle fluctuations (*p* = 0.001), fewer fluoroscopy exposures (*p* = 0.044), and shorter prosthesis implantation times (*p* = 0.017). The retractor group received significantly larger prostheses (*p* < 0.001). Postoperatively, the retractor group exhibited greater FSU height, disc height, and larger interfacet distance (all *p* < 0.005) but these differences diminished by 6 months. The retractor group also showed smaller bone–implant interface gap (*p* = 0.003) at 1 week, and more segmental range of motion (*p* < 0.008) and FSU height loss (*p* = 0.002) at 6 months. Subsidence and heterotopic ossification rates were comparable between groups.

**Conclusion:**

During Prestige LP implantation in CDA, retaining the Caspar retractor enhanced intraoperative stability, improved prosthesis placement accuracy, and reduced fluoroscopy use, achieving comparable clinical outcomes. While associated with greater FSU height loss at 6 months, it did not increase complications, supporting its viability as a standard technical modification for Prestige LP implantation.

## Graphical abstract

This study evaluated the effect of retaining the Caspar retractor during Prestige LP implantation in cervical disc arthroplasty (CDA). Although the manufacturer recommends removal, removal caused vertebral instability during endplate drilling/cutting. Retaining the retractor reduced angle fluctuations, implantation time, and fluoroscopy usage, though taller prostheses were used in the retractor group. At 6 months postoperatively, outcomes were comparable between the two groups, with the retractor group showing greater FSU height loss but similar subsidence rates. These findings offer an optional technique and highlight the need for long‐term follow‐up.

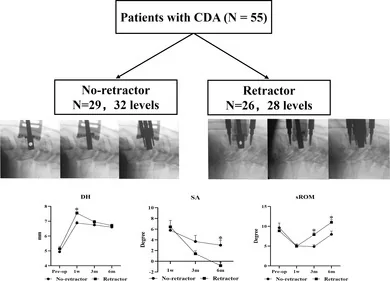

## 1 Introduction

Cervical disc arthroplasty (CDA) is an effective treatment for degenerative cervical radiculopathy and/or myelopathy [1]. In indicated patient populations, CDA yields clinical outcomes comparable to anterior cervical discectomy and fusion (ACDF) while preserving segmental mobility [2]. In CDA, distraction is a critical step after discectomy for adequate intervertebral space exposure, greatly facilitating precise endplate manipulation and effective neural decompression [3]. However, in the surgical technique guide for the Prestige LP artificial disc, the retractor was then relaxed or completely removed before implantation [4]. This recommendation appears to primarily reflect concerns that the retractor may interfere with specialized instrumentation required for rail channel drilling and cutting. In practice, we observed that retractor removal introduced vertebral instability during the critical steps of endplate modification and prosthesis insertion, potentially compromising precision and increasing operative complexity. Our attempt to retain the Caspar retractor showed improvements in disc space stability and procedural efficiency. This technical modification further enhances prosthetic implantation accuracy and surgical control by maintaining stable disc space distraction.

Prestige LP has demonstrated high success rates and patient satisfaction in 10‐year follow‐ups for single‐ and double‐level surgeries [5, 6]. It is also one of the only three FDA‐approved prostheses for double‐level cervical disc replacement. The role of sustained distraction during cervical disc arthroplasty remains controversial. Proponents argue that maintaining intervertebral distraction facilitates precise endplate preparation, ensures optimal prosthesis positioning, and maximizes neural decompression [7]. Conversely, concerns have been raised that excessive or prolonged distraction may lead to facet joint over‐distraction, resulting in persistent postoperative neck pain, accelerated facet joint degeneration, or selection of oversized prostheses with subsequent reduction in segmental mobility [8, 9]. However, these arguments are primarily based on biomechanical hypotheses rather than clinical comparative data. For the Prestige LP system specifically, whether the benefits of enhanced stability outweigh the theoretical risks of over‐distraction has not been empirically tested.

Therefore, this study was designed to provide direct clinical evidence comparing intraoperative stability, radiographic outcomes, and clinical efficacy between the standard technique (retractor removal per manufacturer guidelines) and a modified technique (sustained retractor retention) during Prestige LP implantation.

## 2 Methods

### 2.1 Participants and Inclusion/Exclusion Criteria

Between January and December 2025, patients who underwent CDA, using the Prestige LP artificial disc (Medtronic Sofamor Danek USA Inc.) by the same experienced spinal surgeon were retrospectively reviewed. This study was approved by the Institutional Ethics Committee of our institution (approval No. 2025‐1255). All patients provided written informed consent for follow‐up and the use of their clinical and radiographic data.

Inclusion criteria were cervical spondylosis treated with CDA, a minimum of 6 months follow‐up, and clear intraoperative fluoroscopy and lateral radiographs. Exclusion criteria included trauma, infections, tumors, osteoporosis, or ossification of the posterior longitudinal ligament (OPLL). Additional exclusions were severe facet joint degeneration, disc range of motion < 2°, segmental instability with translational displacement > 3 mm, > 50% loss of intervertebral height, or revision surgery.

### 2.2 Surgical Procedure

The surgical procedure employed a right anterior cervical approach. Following complete discectomy, the Caspar retractor was carefully positioned and gradually distracted to achieve optimal intervertebral exposure. Thorough decompression and excision of nucleus pulposus and cartilaginous endplate were carried out at all targeted levels. In the no‐retractor group, the Caspar retractor was removed after preliminary endplate preparation. Subsequent prosthesis placement steps (endplate rasping, rail channel drilling, rail cutting, prosthesis implantation, and final verification) were completed without the retractor. In the retractor group, the retractor was retained and adjusted to an appropriate tension before proceeding with the same steps. Fluoroscopy was performed after each step for confirmation. In both groups, an appropriate prosthesis size was selected by trial. A drainage tube was inserted after hemostasis before wound closure. The drain was removed 2 days after surgery, and early ambulation was encouraged. Patients with single‐ or double‐level CDA were instructed to wear a hard neck collar for 2 weeks before neck exercises.

### 2.3 Clinical and Radiographic Evaluation

Patients were evaluated preoperatively, 1 week, 3 months, and 6 months postoperatively. Clinical outcomes were assessed using the Japanese Orthopedics Association (JOA) score, Visual Analogue Scale (VAS), and Neck Disability Index (NDI).

Lateral neutral and flexion‐extension radiographs were collected. Intraoperative lateral fluoroscopy images were also collected, covering six surgical steps: trial implantation, endplate rasping, rail channel drilling, rail cutting, prosthesis implantation, and final verification. If multiple fluoroscopy images were taken for the same step, the last one was selected for measurement.

**FIGURE 1:**
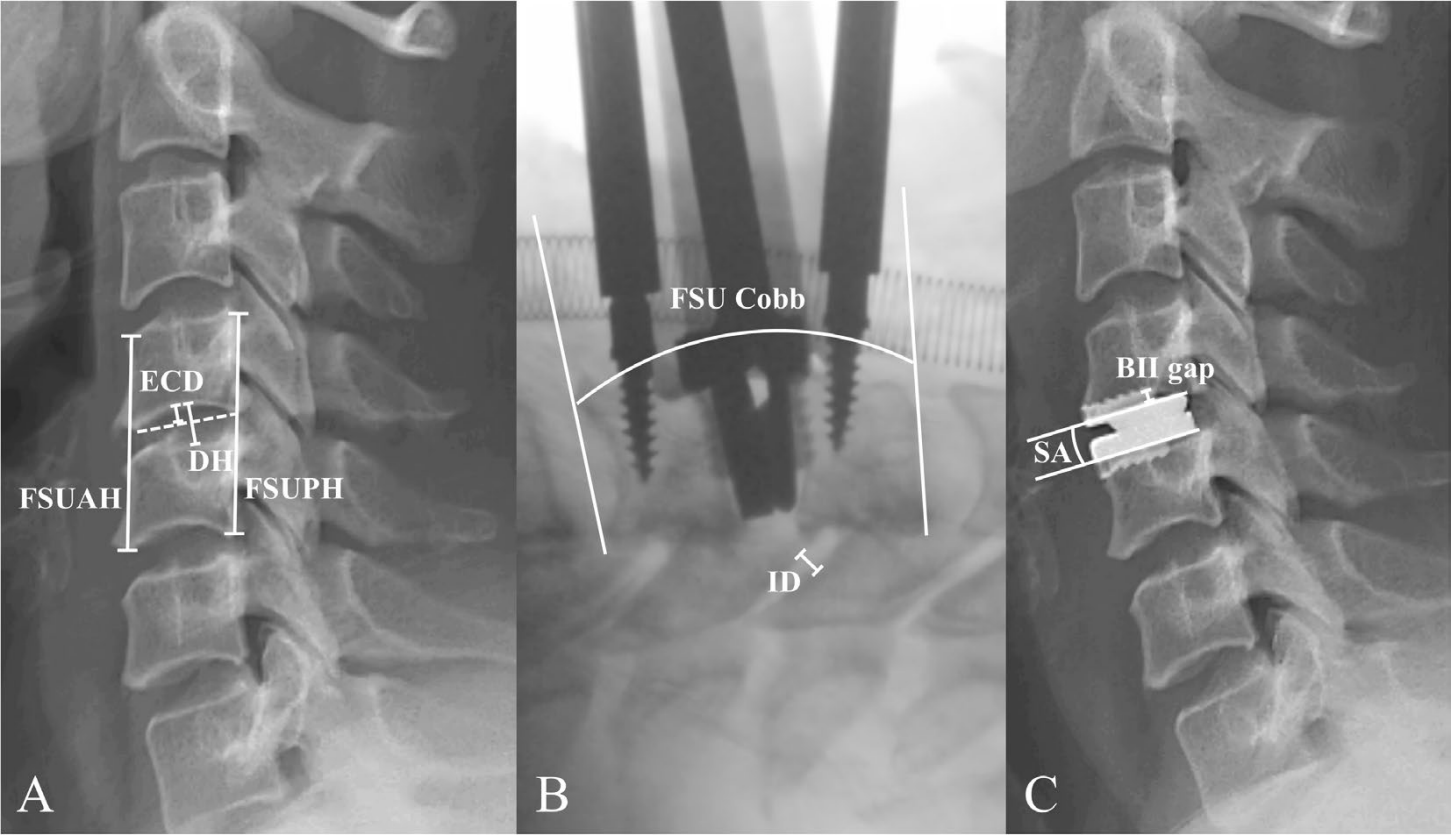
Radiological parameter assessment. ECD and DH were measured by the same point of apex. The two were intentionally misaligned in this visualization for illustrative clarity. BII gap, bone–implant interface gap; DH, disc height; ECD, endplate concave depth; FSU Cobb, functional spinal unit Cobb angle; FSUAH, functional spinal unit anterior height; FSUPH, functional spinal unit posterior height; ID, interfacet distance; SA, shell angle.

Radiographic parameters included length and angle parameters (Figure 1). Length parameters comprised the anterior and posterior height of the functional spinal unit (FSUAH and FSUPH), disc height (DH), bone–implant interface gap (BII gap), endplate concave depth (ECD), and interfacet distance (ID). DH was defined as the distance between superior endplate concave apex and inferior endplate. BII gap was evaluated between the prosthesis upper footplate and vertebra superior endplate [10]. ECD was the perpendicular distance from the concave apex of the superior endplate to the line connecting the anterior and posterior edges [11]. ID was the distance between the facet joint surfaces [12]. Angle parameters included the Cobb angle of the functional spinal unit (FSU Cobb), shell angle (SA) between the two footplates of the prosthesis, and surgical segmental range of motion (sROM) as the difference in FSU Cobb between flexion and extension radiographs. The inferior endplate configuration of the superior vertebra was visually divided into three types according to where the endplate curve apex was situated: posterior (Type I), central (Type II), or anterior (Type III). Lengths were calibrated using known distances from surgical instruments and prosthesis. The timing of fluoroscopy of each step was recorded to calculate step durations. For multiple images, the last image timing was used. All radiographic parameters were measured by two independent spine surgeons blinded to group allocation using ImageJ 1.52 (National Institutes of Health, USA), who underwent standardized training prior to measurement. Each observer measured each parameter twice, with a 2‐week interval between repeated measurements, and the mean of the two measurements was used for final analysis. Intra‐ and interobserver reliability were assessed using the intraclass correlation coefficient (ICC), with intraobserver ICCs ranging from 0.89 to 0.97 and interobserver ICCs ranging from 0.87 to 0.98, indicating excellent measurement consistency (see Table S1).

Complications were also recorded. Subsidence was defined as an average loss of FSUAH and FSUPH > 3 mm at the final follow‐up [13]. Heterotopic ossification (HO) was classified using McAfee's classification (Grades I–IV) [14].

### 2.4 Statistical Analysis

Statistical analysis was conducted using SPSS (version 27, IBM SPSS Inc.). Continuous data were summarized as mean ± standard deviation, and categorical data as counts and proportions. Two‐way repeated measures ANOVA (with group and time as factors) was used to compare longitudinal trends between groups. Pairwise comparisons were performed if ANOVA detected significant differences, with significance thresholds adjusted using the Bonferroni method. Besides ANOVA, comparisons were performed using independent *t*‐test, Mann–Whitney *U* test, or paired *t*‐tests as appropriate. Proportions were compared using the Chi‐square test or Fisher's exact test, and ordinal categorical variables were analyzed with the Mann–Whitney *U* test. A two‐sided *p*‐value < 0.05 was considered statistically significant.

## 3 Results

### 3.1 Patient Demographics

A total of 55 patients were analyzed, including 29 patients (32 segments) in the no‐retractor group and 26 patients (28 segments) in the retractor group. Of these, 28 (50.9%) were male, and 27 (49.1%) were female. The mean age at surgery was 45.93 ± 5.93 years. Procedures included 50 single‐level CDA and 5 double‐level CDA. Baseline data were comparable between groups. The mean height of prosthesis was significantly greater in the retractor group (*p* < 0.001), and the distribution of height sizes differed between groups (*p* < 0.001) (Table 1).

**TABLE 1:** Demographic and surgical data of patients.

Characteristics	No‐retractor	Retractor	*t*/*χ* ^2^/*z*	*p*
No. of patients (*n*)	29	26		—
No. of levels (*n*)	32	28		—
Gender (M/F)^b^	15/14	12/14	0.172	0.680
Age (years)^a^	47.4 ± 5.3	45.1 ± 6.0	1.523	0.140
BMI (kg/m^2^)^a^	24.1 ± 2.7	23.2 ± 3.5	1.067	0.266
Surgical procedure (%)^b^			0.160	0.792
Single‐level CDA	26 (89.7%)	24 (92.9%)		—
Double‐level CDA	3 (10.3%)	2 (7.1%)		—
Arthroplasty level (%)^b^			1.683	0.641
C3/4	2 (6.3%)	1 (3.6%)		—
C4/5	12 (37.5%)	9 (32.1%)		—
C5/6	11 (34.4%)	14 (50.0%)		—
C6/7	7 (21.8%)	4 (14.3%)		—
Endplate type (%)^b^			0.734	0.699
Type I	9 (28.1%)	10 (35.7%)		—
Type II	16 (50.0%)	11 (39.3%)		—
Type III	7 (21.9%)	7 (25.0%)		—
Operation time (min)^a^	156.3 ± 22.5	143.3 ± 27.2	1.936	0.067
Blood loss (mL)^a^	114.3 ± 35.6	108.3 ± 19.0	0.772	0.467
Follow‐up time (months)^a^	6.5 ± 0.8	6.7 ± 0.9	−0.881	0.366
Implant height (mm)^a^	5.91 ± 0.69	6.57 ± 0.50	−4.009	**< 0.001**
Implant size (%)^b^			15.506	**< 0.001**
5 mm	9 (28.1%)	0 (0%)		—
6 mm	17 (53.1%)	12 (42.9%)		—
7 mm	6 (18.8%)	16 (57.1%)		—

*Note:* Bold values are statistically significant.

Abbreviations: BMI, body mass index; CDA, cervical disc arthroplasty.

^a^ Independent *t*‐test.

^b^ Chi‐square test.

### 3.2 Clinical Outcomes

Both groups showed statistically significant recovery in JOA, NDI, and VAS scores at the 6‐month evaluation (*p* < 0.05 for all within‐group comparisons). Intergroup analysis demonstrated comparable efficacy (all *p* > 0.05) (Table 2).

**TABLE 2:** Comparison of clinical outcomes and complications between groups.

	No‐retractor	Retractor	*t*/*χ* ^2^/*z*	*p*
JOA^a^				
Pre‐op	11.5 ± 2.3	11.7 ± 3.3	−0.258	0.809
6 m	15.8 ± 1.7	16.2 ± 3.1	−0.584	0.566
NDI^a^				
Pre‐op	28.2 ± 4.7	27.5 ± 3.0	0.665	0.509
6 m	7.0 ± 3.1	7.5 ± 4.4	−0.482	0.632
VAS^a^				
Pre‐op	6.5 ± 1.2	6.8 ± 1.5	−0.813	0.420
6 m	1.4 ± 1.0	1.3 ± 0.9	0.390	0.697
Subsidence (%)^b^	3 (9.4%)	4 (14.3%)	0.035	0.695
HO formation (%)^b^	15 (46.9%)	10 (35.7%)	0.765	0.382
HO classification (%)^b^			2.273	0.267
Grade I	12 (80.0%)	10 (100%)		
Grade II	3 (20.0%)	0 (0%)		
FSU height loss^a^	0.83 ± 1.12	1.79 ± 1.18	−3.219	**0.002**

*Note:* Bold values are statistically significant.

Abbreviations: FSU, functional spinal unit; HO, heterotopic ossification; JOA, Japanese Orthopedic Association; NDI, Neck Disability Index; VAS, Visual Analogue Scale.

^a^ Independent *t*‐test.

^b^ Chi‐square test.

### 3.3 Functional Spinal Unit: Cobb Angle and Heights

**FIGURE 2:**
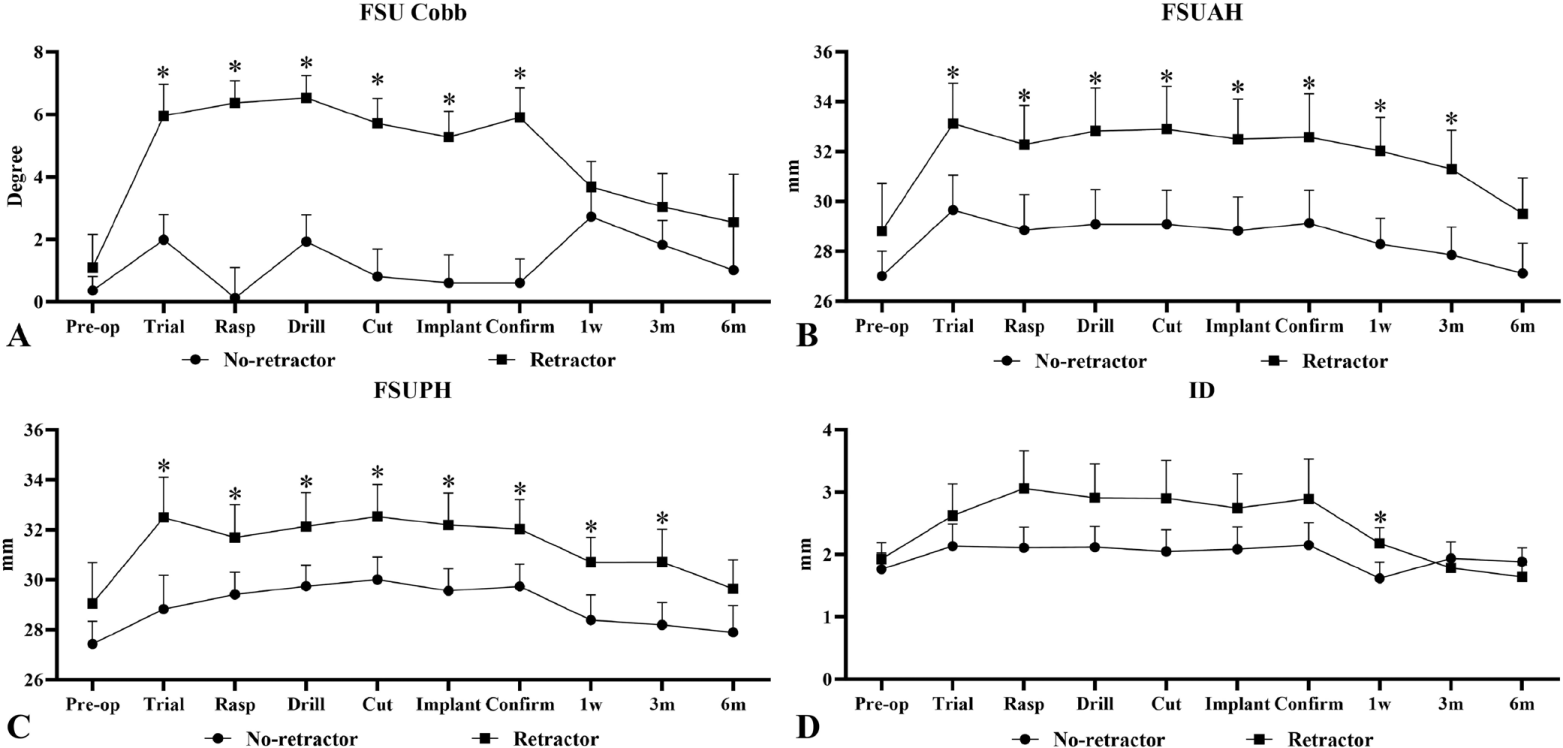
Longitudinal changes of FSU and facet parameters. Asterisks (*) indicate significant intergroup difference (2‐tailed *p* < 0.05). (A) The retractor group exhibited higher and more stable FSU Cobb angles intraoperatively. The no‐retractor group experienced angle fluctuation primarily between the trialing and cutting steps. (B) and (C) FSUAH and FSUPH increased intraoperatively but decreased in follow‐up. The retractor group showed significantly higher values both during and 3 months after surgery. (D) A significant intergroup difference in ID was observed only at 1 week postoperatively. FSU Cobb, functional spinal unit Cobb angle; FSUAH, functional spinal unit anterior height; FSUPH, functional spinal unit posterior height; ID, interfacet distance.

The FSU Cobb increased postoperatively and then declined during follow‐up, while intraoperative trends differed between groups (*p* = 0.001). The no‐retractor group exhibited greater fluctuations during the first four steps (Figure 2A). In intergroup comparisons, intraoperatively, the retractor group had significantly larger Cobb angles (all *p* < 0.005). Longitudinally, no difference was detected before surgery and during follow‐up (Table S2).

FSUAH and FSUPH increased intraoperatively and gradually decreased during follow‐up. In intergroup comparisons, at intraoperative, 1‐week, and 3‐month time points, the retractor group had significantly greater heights (all *p* < 0.005). No significant differences were found between preoperative and 6‐month heights in either group (Figure 2B,C, Table S2).

### 3.4 Facet Joint: Interfacet Distance

Both groups exhibited an intraoperative increase in ID and postoperative decline. Significant intergroup difference was detected only at 1 week postoperatively, with the retractor group showing greater distances (*p* = 0.002). There were no significant differences between pre and post operation time points (Figure 2D, Table S3).

### 3.5 Prosthesis and Endplate: Bone–Implant Interface Gap, Endplate Concave Depth, Disc Height, Shell Angle, and Segmental Range of Motion

Parameters related to the prosthesis and endplate were measured before and/or after surgery. The BII gap was measured at 1 week postoperatively and compared with the ECD, as the BII gap represents the residual ECD. In both groups, the BII gap was significantly smaller than the ECD (all *p* < 0.001). The BII gap was smaller in the retractor group (*p* = 0.004), while preoperative ECD showed no significant difference (*p* = 0.062).

**FIGURE 3:**
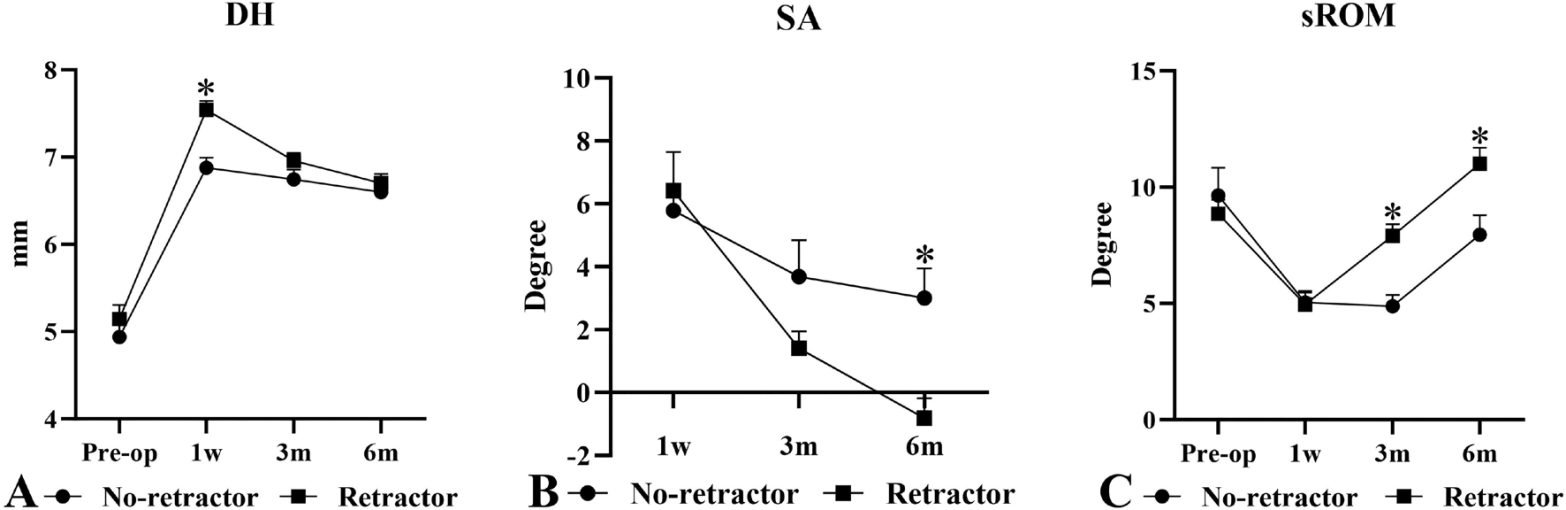
Disc and prosthesis parameter changes before and/or after surgery. Asterisks (*) indicate significant intergroup difference (2‐tailed *p* < 0.05). (A) Surgery recovered DH significantly. DH intergroup difference increased to significance at 1 week after surgery but did not last at later time points. (B) SA decreased postoperatively, showing a statistically significant intergroup difference at 6 months. (C) Both groups demonstrated reduced sROM at 1 week postoperatively; however, the retractor group showed faster recovery and greater range of motion than the no‐retractor group at 6‐month follow‐up. DH, disc height; SA, shell angle; sROM, segmental range of motion.

For DH, intergroup differences were significant only at 1 week postoperatively (*p* < 0.001). DH remained higher than preoperative values at all follow‐up time points in both groups (all *p* < 0.001) (Figure 3A, Table 3).

**TABLE 3:** Comparison of prosthesis and endplate radiographic parameters between groups.

	No‐retractor	Retractor	*t*/*χ* ^2^/*z*	*p*
ECD (mm)	1.79 ± 0.30	1.62 ± 0.36	1.995	0.062
BII gap (mm)	0.79 ± 0.38	0.47 ± 0.45	2.988	**0.003**
DH (mm)^a^				
Pre‐op	4.94 ± 0.83	5.15 ± 0.84	−0.972	0.336
1 week	6.87 ± 0.67	7.54 ± 0.56	−4.169	**< 0.001**
3 months	6.74 ± 0.66	6.95 ± 0.48	−1.392	0.148
6 months	6.60 ± 0.49	6.70 ± 0.56	−0.738	0.448
SA (degree)^b^				
1 week	5.79 ± 3.73	6.42 ± 5.31	−0.537	0.603
3 months	3.69 ± 5.27	1.40 ± 2.32	2.125	0.032
6 months	3.01 ± 5.03	−0.81 ± 3.13	3.472	**0.001**
sROM (degree)^a^				—
Pre‐op	9.65 ± 6.70	8.86 ± 3.20	0.569	0.555
1 week	5.05 ± 2.77	4.95 ± 2.72	0.141	0.892
3 months	4.88 ± 2.75	7.91 ± 2.67	−4.315	**< 0.001**
6 months	7.95 ± 4.78	11.00 ± 3.64	−2.749	**0.008**

*Note:* Bold values are statistically significant.

Abbreviations: BII, bone–implant interface; DH, disc height; ECD, endplate concave distance; Pre‐op, pre‐operation; SA, shell angle; sROM, segmental range of motion.

^a^ Bonferroni method adjusted threshold *p* = 0.0125.

^b^ Bonferroni method adjusted threshold *p* = 0.0167.

SA was measured only postoperatively. Two groups were significantly different only at 6 months (*p* = 0.001), with the retractor group showing smaller SA. After 6 months, SA was significantly lower than that at 1 week in both groups (both *p* < 0.001) (Figure 3B, Table 3).

In both groups, sROM decreased, but bounced back after 3 months. The retractor group exhibited earlier recovery (*p* = 0.022). No significant differences were detected between preoperative and 6‐month. At 3 and 6 months postoperatively, the retractor group had significantly greater sROM than the no‐retractor group (both *p* < 0.008) (Figure 3C, Table 3).

### 3.6 Step Duration and Fluroscopy Usage

The durations of four steps (endplate rasping to prosthesis implantation) and total time were analyzed. The retractor group had significantly shorter time only in the step of prosthesis implantation, saving an average of 49.22 s (*p* = 0.017), and 0.375 time in the number of fluoroscopy times (*p* = 0.044) (Table 4).

**TABLE 4:** Comparison of procedures duration between groups.

	No‐retractor	Retractor	*t*/*χ* ^2^/*z*	*p*
Total time	14:00 ± 04:13	12:39 ± 03:10	1.386	0.174
Rasp	02:38 ± 01:52	02:34 ± 01:24	0.155	0.874
Drill	03:15 ± 01:03	03:03 ± 00:57	0.769	0.473
Cut	03:13 ± 02:25	02:58 ± 00:46	0.524	0.581
Implant^a^	04:53 ± 01:37	02:57 ± 00:45	**2.382**	**0.017**

*Note:* Bold values are statistically significant. All time reported in minutes: seconds format.

^a^ Mann–Whitney *U* test; other comparisons by independent *t*‐test.

### 3.7 Complications

The retractor group exhibited significantly greater FSU height loss at 6 months (mean difference: 0.95 mm, *p* = 0.002). Subsidence was detected in 3 segments (9.4%) of the no‐retractor group and 4 segments (14.3%) of the retractor group (*p* = 0.695). For HO, the no‐retractor group had 15 segments (46.9%) with HO (3 Grade II, 12 Grade I), while the retractor group had 10 segments (35.7%) with HO (all Grade I). No differences were found in incidence (*p* = 0.382) or grading (*p* = 0.267). No loosening, migration, or fractures occurred. No revision was performed (Table 2).

## 4 Discussion

Technical details of CDA are considered critical for outcomes [15]. Regarding retractor use, cautious views exist due to concerns that improper use may lead to facet joint over‐distraction, causing persistent neck pain [16], or oversized prosthesis selection, followed by reduced mobility [17]. However, clinical evidence remained insufficient. Our study demonstrated that retaining the Caspar retractor during Prestige LP prosthesis placement significantly enhanced intraoperative stability, improved prosthetic implantation accuracy, and reduced fluoroscopy use. Specifically, it reduced intraoperative vertebral angle fluctuations, increased the fit between the prosthesis and the bony endplate (as evidenced by a significantly reduced BII gap), and improved procedural efficiency with shorter prosthesis implantation times. At 1 week postoperatively, the retractor group exhibited greater intervertebral and FSU heights and larger interfacet distances, though these differences diminished by 6 months. The retractor group also had smaller shell angles and greater segment mobility at 6 months. While retractor use led to more FSU height loss, no difference was detected in subsidence or HO rates, and clinical outcomes were comparable in the two groups.

### 4.1 Distraction During Artificial Cervical Disc Implantation

Modern artificial cervical discs have diverse designs, while the replacement surgeries generally follow similar steps: discectomy, endplate preparation, trial implantation, and final prosthesis implantation [18]. After discectomy, intervertebral distractors are often applied to separate endplates. The design of these distractors resembles lamina spreaders [19] or Cloward vertebra spreaders [20], but features smooth, toothless tips with broader and flatter profiles. A retainer (Caspar retractor) is additionally applied to maintain this separated position, ensuring stable contact between endplates and the implant during insertion. The technical guidelines by manufacturers of prostheses like Mobi‐C and Baguera C recommend maintaining the retractor until final implantation is completed, while in the Prestige LP guide, the Caspar retractor is loosened or removed before the following steps.

**FIGURE 4:**
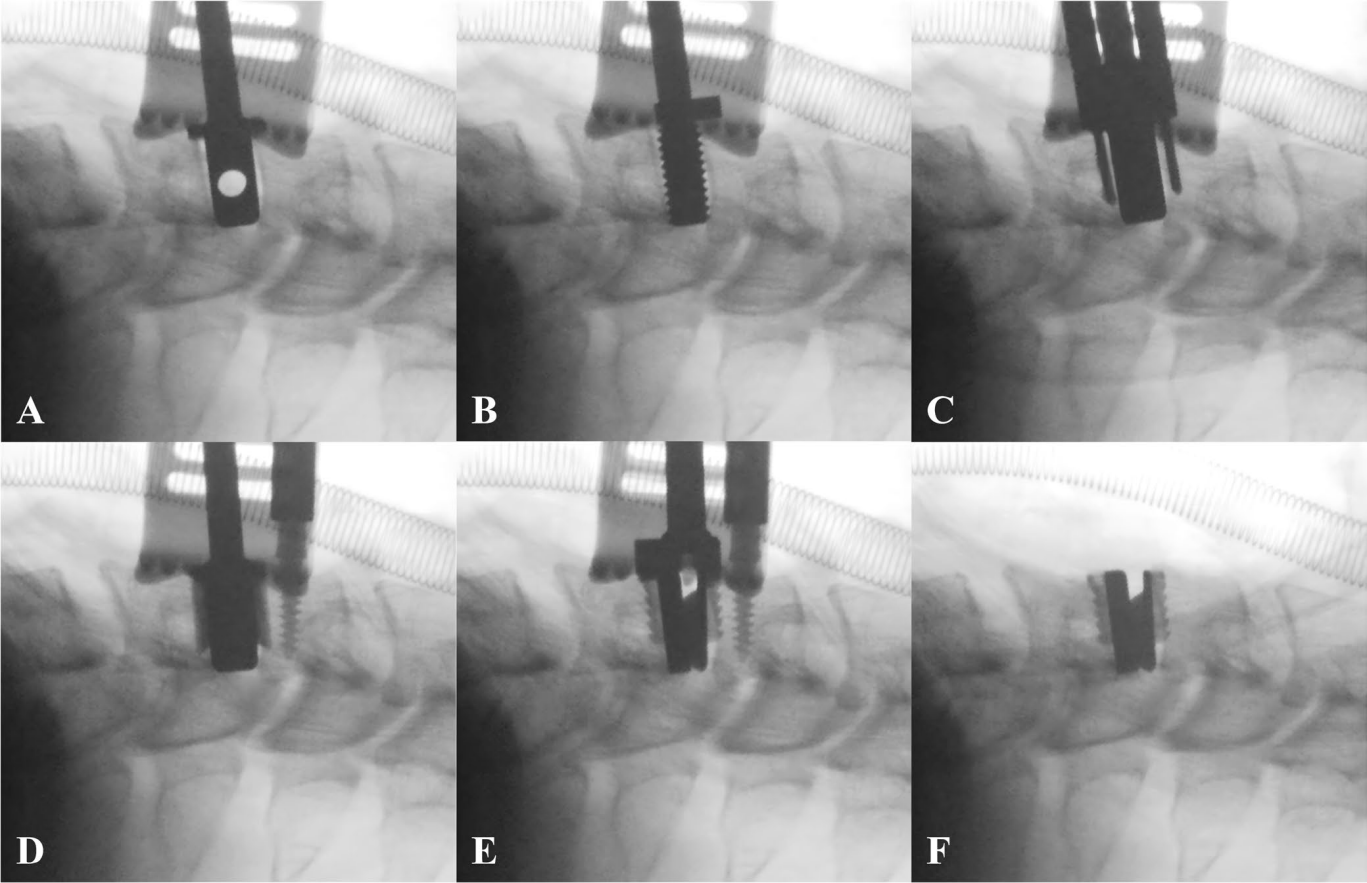
Intraoperative fluoroscopy of prosthesis implantation without retractor use. (A) Acceptable vertebral alignment during trial implantation. (B) Posterior displacement of the superior vertebra following rasping. (C) Further posterior displacement after drilling. (D) Alignment restoration during cutting, with a single retractor pin placed in the superior vertebra for orientation. (E) Prosthesis insertion showing suboptimal superior endplate‐prosthesis gap and lack of posterior contact. Total procedure time from (A) to (E) was 12 min and 41 s, with the prosthesis implantation phase (D) to (E) accounting for 4 min and 53 s.

**FIGURE 5:**
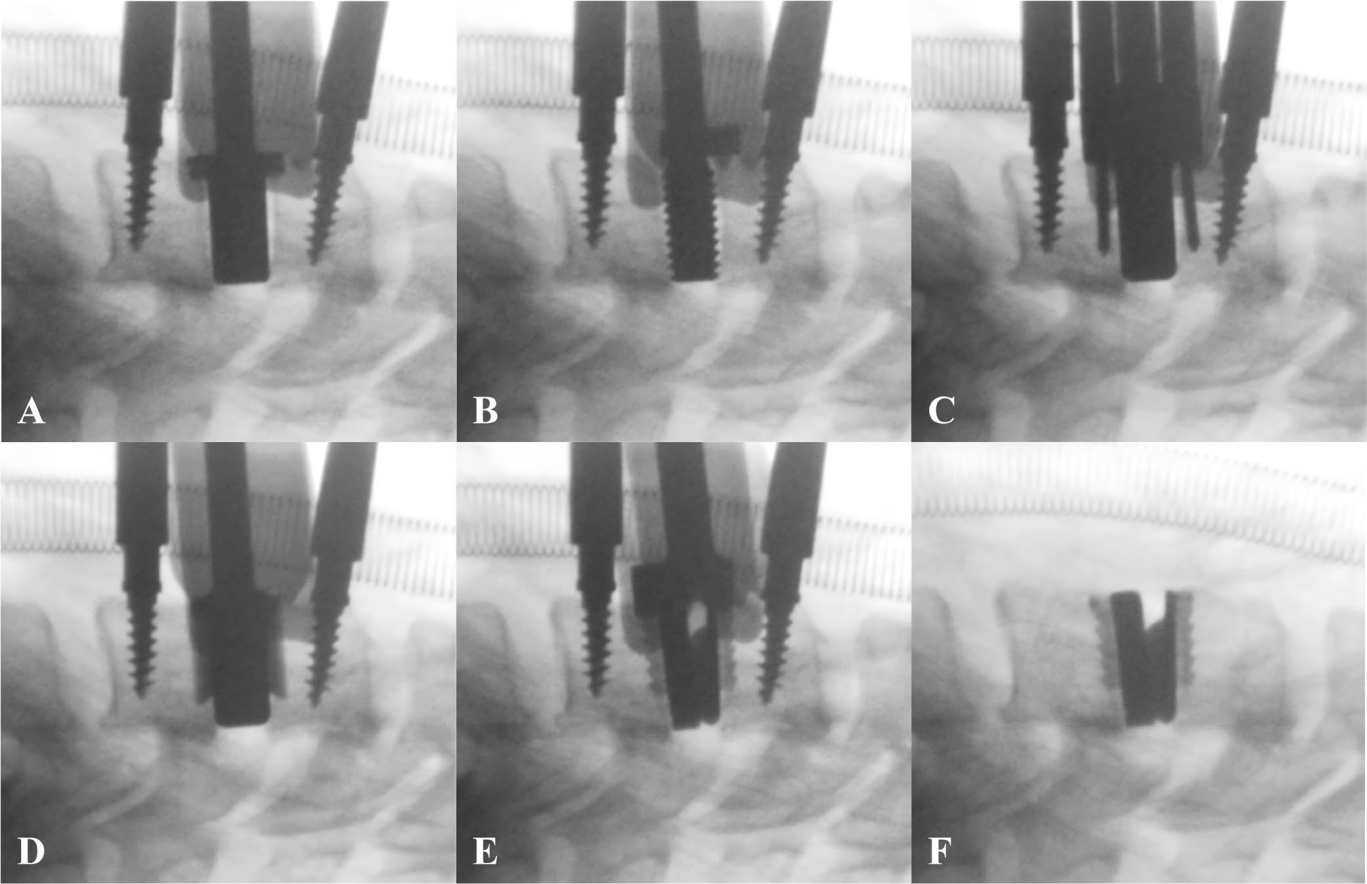
Intraoperative fluoroscopic sequence of prosthesis implantation using Caspar retractors. Changes in intervertebral disc space height during procedural steps (A–E), but with maintained vertebral alignment and stable intervertebral angles throughout the procedure. Total procedure time from (A) to (E) was 11 min and 6 s, with the prosthesis implantation phase (D) to (E) accounting for 3 min and 3 s.

For Prestige LP, the implantation requires significant modification of the endplate morphology and necessitates substantial tapping forces during insertion [21]. In our practice, we observed vertebral displacement and deviation during tapping, leading to a risk of suboptimal drilling, cutting, and implantation, with repeated adjustments and fluoroscopy confirmation, which inspired our modification to keep distraction (Figure 4). Retaining the Caspar retractor mitigated these issues. First, it provided a stable surgical field, enabling more precise endplate preparation and more controlled prosthesis insertion. During cutting, the cutting force itself played the primary role in a stable environment, rather than relying on passive vertebral displacement to achieve effective channel creation [22]. However, in some patients with sclerotic or hard endplate bone, cutting easily caused posterior vertebral displacement, resulting in insufficient effective endplate preparation; in others, despite no obvious vertebral instability on preoperative imaging, peri‐vertebral soft tissue laxity similarly made the cutting and prosthesis insertion process difficult to complete efficiently. Under these circumstances, surgeons often required multiple adjustments to achieve satisfactory implantation conditions [23]. Retaining the Caspar retractor enabled sustained and stable disc space distraction, providing stable operative support for cutting and prosthesis insertion, thereby reducing intraoperative angle fluctuations, improving prosthesis‐to‐bone endplate apposition, and enhancing implantation accuracy. This indicates that the technique offers an effective alternative for reducing the frequency of intraoperative adjustments (Figure 5). It should be noted that fluoroscopic imaging is static, whereas the actual displacement under dynamic surgical manipulation may be substantially greater, particularly during cutting and insertion where repeated fluoroscopic confirmation is frequently required. The reduction in fluoroscopy usage (0.375 fewer exposures per procedure, *p* = 0.044) carries clinical significance beyond statistical significance. Each fluoroscopic exposure during cervical surgery contributes to radiation exposure to the surgeon's hands and the patient's thyroid gland [24]. Over a surgeon's career, cumulative radiation reduction may yield occupational health benefits. Moreover, fewer fluoroscopy shots indicate fewer intraoperative adjustments, suggesting that the modified retractor‐assisted technique provides more predictable and controlled implantation conditions under stable disc space distraction.

Additionally, the smaller BII gap in the retractor group may benefit from deeper and more precise cutting due to stabilized vertebrae. This may also explain the time saved during prosthesis implantation, as precise cutting facilitates easier positioning of the prosthesis into its predetermined location. A reduced BII gap indicates better fit between the prosthesis and the bony endplate, and previous studies have suggested that a smaller gap may contribute to decreased HO formation in the long‐term [10, 25]. While HO incidence did not differ significantly between groups (35.7% vs. 46.9%, *p* = 0.382), the distribution favored the retractor group, with no Grade II HO cases compared to three in the no‐retractor group. Although this difference was not statistically significant—likely due to limited statistical power with 60 segments total—the trend is biologically plausible. The smaller BII gap in the retractor group (0.47 mm vs. 0.79 mm, *p* = 0.003) may reduce micromotion at the bone–implant interface, a known stimulus for HO formation [26]. Alternatively, more precise endplate preparation with stabilized vertebrae may improve osteointegration and reduce reactive bone formation [27].

### 4.2 Prosthesis Size Selection and Radiographic Implications

The most notable difference between groups was prosthesis height, with the retractor group receiving significantly taller implants (mean difference: 0.66 mm, *p* < 0.001) and a markedly higher proportion of 7 mm prostheses (57.1% vs. 18.8%). This raises two important clinical considerations. First, sustained distraction maintained by the Caspar retractor may lead to selection of oversized prostheses, which has been associated with increased facet joint stress and reduced segmental mobility in some studies [28, 29]. However, our 6‐month data showed comparable or even greater sROM in the retractor group, suggesting that the modest height increase (< 1 mm) did not cause clinically significant over‐distraction or mobility restriction within the current follow‐up period. Although sustained distraction increased the initial disc space height, the smaller BII gap in the retractor group indicates a more precise fit between the prosthesis and the bony endplate, with more uniform stress distribution that avoids the adverse effects of focal stress concentration on mobility [25]. Meanwhile, the load‐sharing mechanism of the mobile prosthesis allows partial load transfer through the facet joints rather than concentrating entirely at the bone–implant interface, which also contributes to maintaining segmental mobility [30]. Therefore, despite taller prostheses, the retractor group demonstrated greater—not restricted—segmental mobility at 6 months postoperatively, likely benefiting from early restoration of stable motion due to superior endplate preparation and better osteointegration. Second, the relationship between prosthesis height and adjacent segment disease remains controversial [31, 32]. While our short follow‐up precludes assessment of this outcome, the comparable clinical scores and absence of increased HO rates. Long‐term surveillance of at least 2 year or more is essential to determine whether the selection of larger prostheses in the retractor group translates into different clinical outcomes [33].

We also observed that the prosthesis angle in the retractor group decreased from 6.42° ± 5.31° at 1 week postoperatively to −0.81° ± 3.13° at 6 months postoperatively, suggesting that some segments developed a loss of lordosis or even slight kyphosis in SA. We believe that this change may be primarily attributed to the combined effects of intraoperative anterior longitudinal ligament tension release and postoperative adaptive bone remodeling at the prosthesis‐endplate interface. First, the distraction of the intervertebral space using a retractor for prosthesis implantation during surgery may reduce anterior longitudinal ligament tension, thereby affecting early local stability restoration of the operated segment [8]. Second, the retractor group had greater postoperative intervertebral disc space height in the early phase, allowing for a larger posterior rotation arc of the prosthesis. With the increase in patients' daily activities postoperatively and the reconstruction of the overall cervical compensatory mechanisms, this may manifest as mild loss of SA lordosis on static upright lateral radiographs [34, 35]. Importantly, although there was a statistically significant difference in SA between the two groups, neither group developed clinical symptoms related to abnormal prosthesis angle (such as axial pain or neurological deterioration), suggesting that negative SA within this range may not have clinical significance. However, we also note that due to the lack of dynamic fluoroscopic angle assessment, this static measurement may underestimate the true mobility of the prosthesis. The trend of SA changes should continue to be monitored in subsequent follow‐ups to clarify its long‐term clinical significance.

### 4.3 FSU Height Loss: Clinical Implications

The significantly greater FSU height loss in the retractor group (1.79 mm vs. 0.83 mm, *p* = 0.002) warrants careful interpretation. Several mechanisms may contribute to this finding. First, sustained distraction during endplate preparation may cause microfractures or subchondral bone compression, accelerating postoperative settling [13]. Second, the larger prosthesis size in the retractor group may distribute forces differently across the endplate interface [29]. Third, the greater initial disc space height achieved with the retractor may simply provide more “room” for subsequent height loss during bone remodeling [9]. Importantly, this height loss did not translate into increased subsidence rates at 6 months (14.3% vs. 9.4%, *p* = 0.695). Biomechanically, several factors may explain why this greater height loss did not translate into increased subsidence. The smaller BII gap in the retractor group (0.47 mm vs. 0.79 mm, *p* = 0.003) indicates a more precise prosthesis‐endplate fit with uniform stress distribution, avoiding focal stress concentration [10]. Additionally, the mobile prosthesis design allows partial load transfer through facet joints and surrounding soft tissues rather than concentrating entirely at the bone–implant interface, providing mechanical buffering [30]. The greater initial disc space height also provides adequate “buffer space” for physiological remodeling, keeping the remaining height above the critical threshold for subsidence [25]. Finally, the observed height loss at 6 months likely represents early adaptive changes between the endplate and implant rather than ongoing axial migration—the latter being the essential characteristic of subsidence [36]. Notably, despite the greater FSU height loss in the retractor group, this degree of FSU height loss did not result in early functional impairment within the current observation window, and its clinical significance may be more nuanced than simple radiographic measurements would imply. However, we caution that subsidence is a time‐dependent phenomenon, and our 6‐month follow‐up may be insufficient to capture late‐onset settling. The reference to implant‐related bone remodeling peaking at 6 months should not be overinterpreted as reassurance; rather, it underscores the necessity of 2‐year or longer follow‐up to determine whether the observed height loss stabilizes or progresses. If the height loss represents ongoing implant settling rather than benign remodeling, it could predispose to late subsidence or altered biomechanics [26].

### 4.4 Prosthesis Height, Disc Height, Facet Joint Distraction, and Their Effects

Kazarian et al.'s systematic review [37] identified prosthesis height as a potential factor in postoperative DH, C2–7 range of motion, HO, and patient‐reported outcomes, noting that the Prestige LP implant was the subject of > 50% of included studies. Our data indicated that retaining the Caspar retractor led to higher (but less than 1 mm) prosthesis implantation; the DH, FSU height, ID, and sROM at 6 months all recovered to preoperative levels, with comparable HO and subsidence rates. Notably, the ID difference observed at 1 week (larger in the retractor group) had resolved by 3 months, suggesting that the facet joint distraction induced by retractor retention is reversible and does not appear to cause persistent facet over‐distraction. This finding directly addresses the theoretical concern that sustained distraction may lead to permanent facet joint pathology. These results seem to suggest that retractor retention does not cause excessive facet distraction, oversized prosthesis implantation, or accompanying adverse outcomes by 6 months. However, longer follow‐up is needed to assess the implications of greater FSU height loss. Postoperative height changes following cervical disc arthroplasty involve complex interactions between implant settling, endplate bone remodeling, and viscoelastic relaxation of peri‐implant tissues. While ACDF‐related height loss has been described [38], the mechanisms in CDA may differ due to the mobile nature of the implant. In a mobile implant, stress distribution and remodeling patterns are distinct from those in a fused segment, which may affect the long‐term behavior of height loss [39]. Our 6‐month data represent an intermediate timepoint; definitive conclusions about the trajectory of height loss require follow‐up to at least 12 months.

## 5 Limitations

Several limitations warrant consideration when interpreting our findings. First, the study was device‐specific; the findings may generalize primarily to Prestige LP‐like cutting and tapping‐based implants, but not necessarily to keel‐based or ball‐and‐socket designs with different insertion mechanics. Second, the sequential cohort design introduces potential temporal confounding and learning curve bias, despite the surgeon's extensive prior experience. Third, the 6‐month follow‐up is insufficient to assess long‐term outcomes including late subsidence, HO progression, adjacent segment disease, and prosthesis longevity. The observed FSU height loss and larger prosthesis selection are radiographic findings whose clinical significance can only be determined through 2‐year or longer follow‐up. The 6‐month outcomes reported in this study, including comparable subsidence and HO rates, are encouraging but do not guarantee equivalent long‐term results. Finally, the single‐center, single‐surgeon, retrospective design limits generalizability; prospective, multi‐center validation with extended follow‐up would significantly strengthen the evidence base.

## 6 Conclusion

Retaining the Caspar retractor during Prestige LP implantation enhanced intraoperative stability, improved prosthesis placement accuracy, and reduced fluoroscopy use, while achieving comparable clinical outcomes to the no‐retractor approach. While it increased FSU height loss, this did not translate to higher subsidence incidence in 6‐month follow‐up. These promising short‐term results warrant continued follow‐up to assess long‐term durability.

## Author Contributions

**Xiaoqiang Zhao:** writing – original draft, methodology, visualization. **Xiao Yang:** methodology, writing – original draft, writing – review and editing. **Minghe Yao:** conceptualization, writing – original draft. **Kangkang Huang:** visualization. **Tingkui Wu:** methodology. **Shihao Chen:** formal analysis. **Yi Deng:** writing – review and editing. **Hao Liu:** formal analysis, resources, supervision. **Beiyu Wang:** conceptualization, resources, writing – review and editing, supervision, funding acquisition.

## Funding

This research was supported by the 1·3·5 project for disciplines of excellence, West China Hospital, Sichuan University (26HXJS018).

## Disclosure

All authors listed meet the authorship criteria according to the latest guidelines of the International Committee of Medical Journal Editors and are in agreement with the manuscript.

## Ethics Statement

This study was approved by our institutional ethics committee (number: 2025‐1255). All participants provided informed consent for the analysis of their clinical data.

## Conflicts of Interest

The authors declare no conflicts of interest.

## Supporting information


**Table S1:** Intraobserver and interobserver reliability assessments between two measurers over a two‐week period.

**Table S2:** Comparison of FSU paraments between groups.

**Table S3:** Comparison of interfacet distance between groups.

## Data Availability

The data that support the findings of this study are available from the corresponding author upon reasonable request.
