# Supplementary material for: Effect of Keeping Caspar Retractor During Prestige LP Cervical Disc Implantation on Intra‐ and Postoperative Outcomes
Source: Orthop Surg. 2026 Sep 24;18(11):2428–39. doi: 10.1111/os.70438 (PMC13602357; doi:10.1111/os.70438)
Supplement: Supplementary file 3 [file OS-18-2428-s003.docx]

**Supplement Table 1.** Intraobserver and interobserver reliability assessments between two measurers over a two-week period.

| **Parameters** | **Intraobserver ICC *** | **Interobserver ICC ^†^** |
| --- | --- | --- |
| Length parameters |  |  |
| FSUAH (mm) | 0.92 | 0.93 |
| FSUPH (mm) | 0.95 | 0.94 |
| DH (mm) | 0.93 | 0.91 |
| BII gap (mm) | 0.89 | 0.87 |
| ECD (mm) | 0.90 | 0.89 |
| ID (mm) | 0.91 | 0.90 |
| Angle parameters |  |  |
| FSU Cobb (°) | 0.97 | 0.98 |
| SA (°) | 0.96 | 0.96 |
| sROM (°) | 0.93 | 0.92 |

* The intraobserver ICC refers to the ICC calculation of two measurers, in which all data from two measurers for two weeks are statistically analyzed. ^†^ The interobserver ICC involves grouping the data from week 1 and week 2 separately, where all data measured by two surveyors each week are statistically analyzed to calculate ICC.

FSUAH, functional spinal unit anterior height; FSUPH, functional spinal unit posterior height; DH, disc height; BII gap, bone-implant interface gap; ECD, endplate concave depth; ID, interfacet distance; FSU Cobb, functional spinal unit Cobb angle; SA, shell angle; sROM, segmental range of motion.
