# Supplementary material for: Effect of Keeping Caspar Retractor During Prestige LP Cervical Disc Implantation on Intra‐ and Postoperative Outcomes
Source: Orthop Surg. 2026 Sep 24:10.1111/os.70438. Online ahead of print. doi: 10.1111/os.70438 (PMC13602357; doi:10.1111/os.70438)
Supplement: Supplementary file 2 [file OS-9999-0-s002.docx]

**Supplement Table 3.** Comparison of interfacet distance between groups

|  | **No-retractor** | **Retractor** | **P** |
| --- | --- | --- | --- |
| ID* |  |  |  |
| Pre-op | 1.76±0.73 | 1.92±0.69 | 0.351 |
| Trial | 2.13±0.98 | 2.62±1.34 | 0.091 |
| Rasp | 2.10±0.93 | 3.06±1.59 | 0.005 |
| Drill | 2.11±0.94 | 2.91±1.44 | 0.012 |
| Cut | 2.04±0.98 | 2.90±1.59 | 0.013 |
| Implant | 2.08±1.01 | 2.74±1.46 | 0.034 |
| Confirm | 2.15±1.01 | 2.89±1.69 | 0.038 |
| 1w | 1.61±0.73 | 2.17±0.66 | **0.002** |
| 3m | 1.93±0.74 | 1.78±0.39 | 0.295 |
| 6m | 1.88±0.62 | 1.64±0.51 | 0.090 |

* Bonferroni method adjusted threshold p=0.005.

**Bold** values are statistically significant.

ID, interfacet distance.
