# Supplementary material for: Effect of Keeping Caspar Retractor During Prestige LP Cervical Disc Implantation on Intra‐ and Postoperative Outcomes
Source: Orthop Surg. 2026 Sep 24:10.1111/os.70438. Online ahead of print. doi: 10.1111/os.70438 (PMC13602357; doi:10.1111/os.70438)
Supplement: Supplementary file 3 [file OS-9999-0-s003.docx]

**Supplement Table 2.** Comparison of FSU paraments between groups

|  | **No-retractor** | **Retractor** | **P** |
| --- | --- | --- | --- |
| FSU Cobb* |  |  |  |
| Pre-op | 0.36±2.55 | 1.09±5.59 | 0.481 |
| Trial | 1.98±4.54 | 5.95±5.35 | **0.002** |
| Rasp | 0.13±5.49 | 6.36±3.76 | **＜0.001** |
| Drill | 1.92±4.84 | 6.53±3.75 | **＜0.001** |
| Cut | 0.80±5.00 | 5.71±4.21 | **＜0.001** |
| Implant | 0.61±5.05 | 5.27±4.38 | **＜0.001** |
| Confirm | 0.61±4.32 | 5.91±5.01 | **＜0.001** |
| 1w | 2.72±5.62 | 3.67±4.28 | 0.445 |
| 3m | 1.83±4.40 | 3.04±5.64 | 0.363 |
| 6m | 1.01±9.27 | 2.54±8.16 | 0.452 |
| FSUAH* |  |  |  |
| Pre-op | 27.01±2.79 | 28.81±5.03 | 0.071 |
| Trial | 29.65±3.97 | 33.12±4.28 | **0.001** |
| Rasp | 28.85±4.01 | 32.27±4.16 | **0.001** |
| Drill | 29.08±3.91 | 32.83±4.54 | **0.001** |
| Cut | 29.09±3.82 | 32.90±4.53 | **＜0.001** |
| Implant | 28.82±3.82 | 32.49±4.24 | **＜0.001** |
| Confirm | 29.12±3.73 | 32.57±4.59 | **0.001** |
| 1w | 28.29±2.93 | 32.03±3.54 | **＜0.001** |
| 3m | 27.85±3.14 | 31.29±4.13 | **＜0.001** |
| 6m | 27.12±3.40 | 29.50±3.80 | 0.009 |
| FSUPH* |  |  |  |
| Pre-op | 27.42±2.58 | 29.05±4.32 | 0.062 |
| Trial | 28.82±3.82 | 32.49±4.24 | **＜0.001** |
| Rasp | 29.41±2.53 | 31.69±3.46 | **0.003** |
| Drill | 29.74±2.38 | 32.13±3.58 | **0.003** |
| Cut | 30.01±2.56 | 32.53±3.38 | **0.001** |
| Implant | 29.56±2.46 | 32.19±3.39 | **0.001** |
| Confirm | 29.73±2.51 | 32.02±3.11 | **0.002** |
| 1w | 28.38±2.83 | 30.70±2.65 | **0.001** |
| 3m | 28.19±2.52 | 30.71±3.44 | **0.001** |
| 6m | 27.89±3.04 | 29.64±3.04 | 0.022 |

* Bonferroni method adjusted threshold p=0.005.

**Bold** values are statistically significant.

FSU, functional spinal unit; FSUAH, functional spinal unit anterior height; FSUPH, functional spinal unit posterior height.
